# Vertebral Algic Syndrome Treatment in Long COVID—Cases Reports

**DOI:** 10.3390/ijerph182111457

**Published:** 2021-10-30

**Authors:** Andrej Džubera, Juraj Chochol, Róbert Illéš, Alica Chocholová, Erika Zemková

**Affiliations:** 1 Department of Neurosurgery, Faculty of Medicine of Slovak Medical University, University Hospital—St. Michal’s Hospital, Satinského 1, 811 08 Bratislava, Slovakia; andrej.dzubera@nsmas.sk (A.D.); robert.illes@nsmas.sk (R.I.); 2 Department of Pediatric Hematology and Oncology, National Institute of Children’s Diseases, Comenius University, Limbova 1, 833 40 Bratislava, Slovakia; alica.chocholova@nudch.eu; 3 Department of Biological and Medical Sciences, Faculty of Physical Education and Sports, Comenius University in Bratislava, Nábrežie Armádneho Generála Ludvíka Svobodu 9, 814 69 Bratislava, Slovakia; erika.zemkova@uniba.sk; 4 Faculty of Electrical Engineering and Information Technology, Sports Technology Institute, Slovak University of Technology, Ilkovičova 3, 812 19 Bratislava, Slovakia

**Keywords:** SARS-CoV-2 infection, back pain, radiculopathy, surgical treatment, long haulers

## Abstract

Though pain is a frequent symptom of long COVID-19, little attention has been paid to vertebral algic syndrome. Therefore, we present the cases reports of two precisely selected physically active patients where vertebral algic syndrome and radiculopathy dramatically worsened in acute SARS-CoV-2 infections. The vertebral pain with radicular irritation was resistant to conservative treatment in chronic post-COVID syndrome. The neurological difficulties corresponded to the radiologic imaging presented on MRI scans. Due to the absence of standard therapeutic guidelines in literature sources, it was decided to provide routine therapeutic procedures. Spinal surgery with radicular decompression was performed within 6 months after acute SARS-CoV-2 infection. This led to the improvement of their neurological status and was in corroboration with decreases of VAS (from 9 to 0 in Patient 1 and from 7 to 1 in Patient 2). Our experience indicates that these patients benefited from the standard neurosurgical radicular decompression, and sufficient pain relief was achieved; nevertheless, the initial trigger of neurological worsening was acute SARS-CoV-2 infection.

## 1. Introduction

The longer the severe acute respiratory syndrome coronavirus 2 (SARS-CoV-2) pandemic drags on, the more different consequences after infection will occur. Long-term sequelae and medical complications occur in coronavirus disease 2019 (COVID-19) survivors. These can last for weeks or even months.

Post-acute COVID-19 is defined as the persistence of symptoms for more than three weeks, and chronic COVID-19 as lasting beyond 12 weeks from the first signs of SARS-CoV-2 infection [1]. The wide variety of symptoms, signs, and abnormal laboratory parameters are exhibit not only in acute [2,3,4,5,6,7,8,9,10,11,12] but also in chronic COVID-19 [13,14,15]. In the post-COVID period the symptoms of acute disease can persist or relapse, can be remitting, and even new symptoms can occur. Patients show virologic recovery and the majority also biochemical and radiological recovery, but clinical recovery is delayed [13,16]. The time lag to full recovery varies, and those not regaining pre-COVID health status have been reported [13,15,17,18].

In the absence of an agreed definition, various terms are used, such as ‘long COVID´ or ´long haulers’, ‘long-term COVID-19 effects’, ‘post-COVID syndrome’, and ‘persistent COVID-19 symptoms’ [13,14,15].

So far, there has not been sufficiently proven evidence of association between the severity of SARS-CoV-2 infection, patient comorbidities, and the development and organ involvement in post-COVID-19 syndrome. It seems that patients with milder acute COVID-19 managed at home and outpatients are more likely to be affected with prolonged symptoms and disabilities than those after severe acute COVID-19 that required intensive unit care [13]. On the contrary, the more severe acute phase may be associated with more severe symptoms in long COVID-19 [19]. Proposed mechanisms of post-COVID-19 syndrome development include direct and indirect tissue damage caused by acute viral infection, and/or autoimmune processes triggered by infection [11,20]. Nevertheless, exact processes of tissue damage are still unknown.

The prevalence of residual symptoms is 10–87% [1,14,18,21]. More than 50 various symptoms were identified, and at least one symptom has been exhibited in 8/10 COVID-19 survivors [14,22,23]. Coronavirus infections are commonly accompanied by pain [18,21,22,24,25]. Among reported areas affected by pain, less attention has been paid to vertebral pain [22]. Acute pain can continue in the post-infection period [26] and can become chronic [5]. The incidence of musculoskeletal pain during the course of acute COVID-19 is estimated to be up to 46.6% (in cervical) and up to 50.7% (in lumbar spine) [26]; its incidence in long COVID-19 has not yet been estimated [1,13,18,21,22].

The incidence of back pain has an increasing longitudinal trend worldwide [27,28,29] and is accentuated in lock-downs [30]. The identification of low back pain as a symptom of long COVID syndrome among SARS-CoV-2 asymptomatic or oligosymptomatic patients is challenging and might lead to decreased sensitivity of researchers for this topic. Based on the high prevalence of musculoskeletal and joint pain [4,13,14,21,22,25,31,32] and nervous system involvement [4,13,14,22,25,33,34,35,36,37,38], vertebral pain and nerve root impairment in long COVID-19 is underestimated.

As evidence-based knowledge in the treatment of long COVID-19 is still being gathered, symptomatic therapies are widely used in general and specialized care [1,39,40,41,42].

Here, we present successful spinal neurosurgery treatment in two patients who exactly identified acute SARS-CoV-2 infection as a trigger factor of development or worsening of vertebral algic syndrome with radiculopathy. Radicular pain continued after viral recovery and was refractory on conservative treatment of chronic long COVID-19.

## 2. Materials and Methods

Two patients (1 woman and 1 man) were followed up for lumbar vertebral algic syndrome associated with radiculopathy during acute COVID-19 infection (RT-PCR proved SARS-CoV-2). Back pain with radicular irritation persisted after virologic and pulmonary recovery and did not reach the pre-COVID baseline status within 6 months from the acute illness onset. Motor deficit was not present. Spinal surgery was indicated and performed for conservative treatment failure. These two patients were precisely selected among patients with persistent back pain with radicular irritation after acute COVID-19. The inclusion criteria were onset/worsening of back pain in acute COVID-19 and persistence after virologic recovery, good adherence to medical guidelines and conservative treatment, the character of their work not affected by pandemic measures, and no history of even minor vertebral trauma. The exclusion criteria were as follows: not proved SARS-CoV-2 infection or only antigene test positivity, history of vertebral trauma previous to COVID-19, change of daily routine towards smartwork, sedentary life style and low physical activity prior to infection, and not satisfactory adherence to medical guidelines and conservative treatment.

Their subjective feelings of pain were evaluated before the infection (retrospectively) and during neurosurgical examination at onset of back pain, after virologic recovery (more than 14 days after positive RT-PCR test for SARS-CoV-2) and after surgery (3 months after operation). The Visual Analogue Scale (VAS) was used. The patients marked scores from 0–10 (from ‘no pain at all’ to ‘my pain is as bad as it could be’). A higher score indicated greater pain intensity [43]. SARS-CoV-2 was confirmed by the RT-PCR method during acute infection at accredited laboratories in Slovakia. Informed consent was obtained from both subjects.

## 3. Cases Report

### 3.1. Patient 1

Patient 1 was a 51 year old woman (weight 64 kg, height 165 cm, BMI 23.51), a former athlete, and physically active [44]. Acute COVID-19 was presented by fatigue, fever, chills, cough, myalgias, and lumbar vertebral algic syndrome. Symptomatic treatment was provided, and no hospitalization was needed. Worsening of low back pain with new radicular irritation of right L5 and S1 nerve roots was presented with acute COVID-19. The motor deficit was not present and there was no history of low back injury. The back difficulties persisted after microbial recovery. Conservative treatment (pain reduction strategies, physiotherapy, and rehabilitation) did not bring pain relief in during the chronic long COVID period [4].

**Figure 1:**
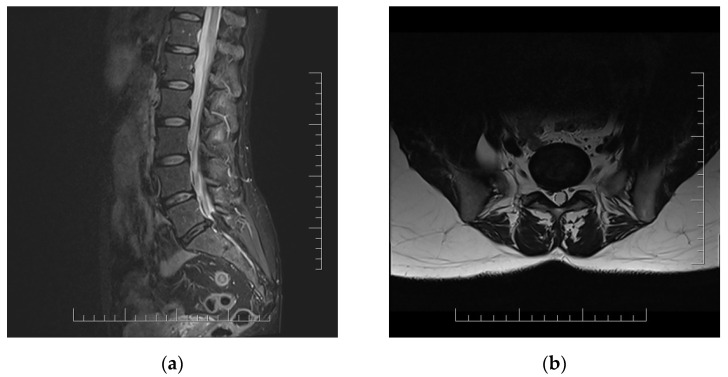
MRI scans of lumbar spine in 51 year old woman with persistent lumbar pain and radicular irritation of right L5 and S1 in chronic long COVID with correlation with L5/S1 disc herniation. (**a**) Sagittal T2-weighted sequence; (**b**) axial T2-weighted sequence.

Lumbar MRI scans were performed twice (2 and 4 months after acute infection). Both MRI scans showed right disc herniation in level L5/S1 in correlation with clinical manifestations (Figure 1).

Surgical treatment was indicated five months after acute COVID-19 infection. Routine spinal operation with radicular decompression of right L5 and S1 nerve root via partial hemilaminectomy of right L5 and S1 vertebras and partial dorsal disc resection were performed. The patient was discharged from the hospital three days after surgery with an appropriate clinical status with no postoperative complications. The pain intensity notably decreased after nerve root decompression and remained stable at the check-up three months after surgery (Table 1). The patient returned to normal life and recreational sport activities.

### 3.2. Patient 2

Patient 2 was a 52 year old man (weight 100 kg, height 180 cm, BMI 30.86), a former athlete, nowadays lightly obese but still physically active [44], was confirmed to be SARS-CoV-2 positive. Acute COVID-19 was manifested by fatigue, headache, fever, chills, myalgias, and low back pain. No history of back injury was present. The mild COVID-19 required only symptomatic treatment in home care.

Patient 2 was an outpatient with pre-infectious vertebral algic syndrome with incipient multiple radiculopathy and sacroiliac joint pain predominantly on the left side. No signs of motor deficit were present. Patient status was compensated by conservative treatment and rehabilitation with no need of surgical intervention. However, the patient´s status worsened dramatically during acute COVID-19 and improved only slightly after microbial recovery. The radicular irritation intensity increased. The radicular pain changed to the opposite side (L3 and L4 nerve root at the right side), and minor lumbar pain persisted. Lumbar MRI scans were performed twice (3 and 5 months after acute infection). Chronic degenerative spine changes with osteochondrosis and spinal stenosis at levels L3/L4 and L4/L5 and predominant foraminal stenosis at right site were identified. The radiological findings were in correlation with the clinical status of the patient.

Spinal surgery was indicated and performed in the sixth month after acute COVID-19 infection. Radicular decompression of the right L3 and L4 nerve root via hemilaminectomy of the L4 vertebra and partial hemilaminectomy of the L3 and L5 vertebras with foraminotomy were performed. The patient was discharged home three days after spinal surgery with no postoperative complications. The pain intensity and root irritation symptoms decreased and remain stabilized three months after surgery (Table 1).

## 4. Discussion

The course of low back pain with radicular irritation in two SARS-CoV-2 positive patients is presented. Both patients reported increased pain intensity in acute COVID-19, which continued after virologic recovery and became chronic. COVID-19 infection was considered as the factor of decompensation of an already existing degenerative spine disorder. After conservative treatment failure, routine spinal operations (radicular decompression via partial or total hemilaminectomy) were performed with satisfactory outcomes. These findings are in agreement with those by Sahin, who reported that half of patients with acute COVID-19 describe musculoskeletal pain in the lumbar spine [26], which continues in the post-infection period and could become chronic [5].

The spine is a complex structure. Vertebral pain genesis in SARS-CoV-2 infection could be provoked by direct and indirect damage mechanisms and autoimmune process triggers [11,20], which could affect muscles, bones, joints, and also neural structures for the neurotropic features of coronaviruses [11,36,37,38]. However, the exact mechanism is not known [11,20].

Nerve root impairment was identified as a gap in the central nervous system COVID-19 manifestation, as attention is predominantly paid to functional and structural brain deterioration [11,31,33,34,35,36,37]. Our clinical praxis indicates that nerve root irritation and back pain presentation in the acute and post-COVID-19 periods are common difficulties in COVID-19 patients. Vertebral pain exhibits various, even bizarre manifestations, which are variable during the course of the disease and which might not be in correlation with radiological findings [12].

About 1/3 of non-severe SARS-CoV-2 infections probably develop long COVID [22], and at least one symptom is present in 80% after recovery from acute SARS-CoV-2 infection [13,14,22]. The identification of long haulers with low back pain is challenging. The reasons for this are manifold. The majority of COVID-19 patients are asymptomatic or oligosymptomatic in the course of acute illness and any type of the novel coronavirus tests might not be performed. The testing strategies vary among countries, and the false negative results might occur in patients when done too early or too late [13,16]. Therefore, distinguishing post-COVID-19 low back pain patients from low back pain patients of different origin could be problematic. The number of patients with low back pain associated with the novel coronavirus infection is assumed to be high but has not been reported in literature so far.

Standard therapeutic procedure guidelines have not been broadly established for long COVID [1,39,40,41,42,43,44], and this is prominent in rare symptoms treatment. The recommendations for low back pain in post-COVID syndrome are absent. Therefore, we proposeto follow routine therapy guidelines and procedures in long COVID patients with back pain and radicular irritation. Satisfactory therapeutic outcomes were achieved in terms of pain relief. The absence of therapeutic guidelines did not enable us to compare selected and recommended procedures.

The findings have to be seen in light of the limitation of small sample size. The identification of patients with low back pain of COVID-19 origin could be challenging, as for example, by the asymptomatic and oligosymptomatic course of acute novel coronavirus infection, SARS-CoV-2 identification might be omitted. The effect of sedentary life changes during lock-downs is also likely to play a role in vertebral pain development.

## 5. Conclusions

Case reports of two patients showed that vertebral algic syndrome with/without radicular irritation may be considered as a symptom of chronic COVID-19. As shown, their vertebral pain with radiculopathy began or increased dramatically during acute SARS-CoV-2 infection. This pain persisted and was resistant to conservative treatment after virologic recovery. Neuroimaging MRI scans were in correspondence with neurological status and complaints. COVID-19 infection was considered as the factor of decompensation of already existing degenerative spine disorder.

Although low back pain in long COVID-19 may be short on evidence for optimal therapy, considerable pain relief was achieved after spinal surgery with radicular decompression performance. This indicates that standard therapeutic procedures provide satisfactory relief in vertebral pain management and increase the quality of life in post-COVID syndrome.

## Figures and Tables

**Table 1:** VAS score in patients prior to, during, and after COVID-19 infection and after surgery.

Patient	Gender	Age (Years)	Pre-COVID-19 VAS	Peri-COVID-19 VAS	Post-COVID-19 VAS	Post-Surgery VAS
1	F	51	1	9	8	0
2	M	52	3	7	5	1

F—female, M—male, VAS—Visual Analogue Scale.

## Data Availability

The data presented in this study are available in the article.
